# Medical Statistics

**Published:** 1837-01

**Authors:** 


### MEDICAL STATISTICS.

*Statistics of Fever in Belfast.* By W. Mateer, m.d., Physician to the Belfast Fever Hospital.

This is a very interesting paper, which we regret that we cannot notice more at length. We must content ourselves with a few of the more important facts contained in it. The following general results apply to a period of eighteen years, viz. from May, 1817, to May, 1835; and are taken from the records of the Fever Hospital. The total number of patients admitted was 11,209, viz. 4,458 males and 5,130 females; and of these, 103 were received in a dying state. The ratio of the deaths to the recoveries was 1 in 15, including the moribund cases, and 1 in 18 excluding these. The average number of days which patients remained in the hospital was twenty-two. In regard to the *causes* of the disease, it would appear that the seasons or period of the year exerted little influence, as the number admitted varied but little in the different quarters. There seems to have been greater difference in the ratio of the mortality, this being in the different seasons as follows: summer, 1 in 17; autumn, 1 in 15; winter, 1 in 14; spring, 1 in 13. The influence of the important cause, *contagion,* is illustrated by a table; but the results can only be admitted as probable, as many will be disposed to disallow the evidence admitted as proof of contagion, viz. the mere fact of *two or more individuals being brought from the same house or family.* Assuming this test of contagion to be legitimate, out of the total admissions, 7,246 originated from this cause, and only 2,342 from causes of a different kind. One of the most striking results is the beneficial influence produced on the curability of fever by early removal to the hospital; a result which has been often observed. The ratio of mortality per cent., according to the period of the disease at the time of admission, is as follows: of the cases admitted on the second day of the disease, the mortality was 2 per cent.; on the third, 3; on the fourth, fifth, and sixth, 4; on the seventh, 6; on the eighth, 11; on the ninth and tenth, 10; on the eleventh, 6; on the twelfth, 10; on the fourteenth, 20.

The most uniform results obtained from any part of Dr. M.'s calculations are these in reference to the influence that *age* has on the number and mortality of fever cases. It would appear that the susceptibility to the disease diminishes, while its fatality increases with the increase of age. Thus, there were admitted from birth to the twentieth year, 5,214 patients, and the mortality was only two per cent.; from twenty to forty, the number was 3,747, and the mortality eight per cent.; from forty to sixty, the number was 1,043, and the mortality twenty per cent.; from sixty to eighty, the number 171, and the mortality thirty-five per cent. The female sex would seem to exert an analogous influence with tender age, giving a greater predisposition to be effected and an inferior mortality: thus, of the total admissions, 5,130 were females and 4,458 males; while the general mortality among the former was 1 in 16, and in the latter 1 in 13. Nothing positive could be ascertained as to the influence of trades in giving rise to fever, as the proportion which the different trades bear to the whole population could not be ascertained.

The following remarks upon the relative prevalence of the disease in different parts of the town are interesting, and the observation respecting the deleterious influence of a deficient supply of water very important, both in a medical and hygienic point of view. "The streets inhabited by the poorer classes supply the greatest share; but they differ greatly in this respect. One street called Carrickhill, and its continuation, Millfield, with the adjoining lanes and entries, are found to have supplied nearly three-fifths of the whole amount; and yet these are by no means the poorest or worst-ventilated parts of the town. The circumstance would seem to be caused by a deficiency in the proper supply of water to this district, from its occupying a higher elevation than the source whence the rest of the town is supplied. Water is procured in other ways, but with difficulty, and not in the quantities they would otherwise have it. The consequences are want of cleanliness and bad sewerage, so that decayed animal and vegetable matter of all kinds, not being carried off by a current of water in the usual way, accumulate, and so generate miasmata." In making this statement, Dr. Mateer does not seem to perceive how much it militates against his opinion of contagion being the great source of fever in Belfast. *Dublin Journal, September,* 1836.

---

*On the comparative Frequency in which the principal Medicines have been prescribed in Dublin, during the last sixty years.* By W. D. MOORE.

MR. MOORE, apothecary, has examined the prescriptions in his establishment, (which was commenced by his grandfather in 1780,) in order to estimate the use made of the principal medicines by the most eminent physicians and surgeons of Dublin. For this purpose, he divided the entire time into three equal portions, and from each of these he took 1,200 prescriptions, and marked the frequency with which each medicine occurred in them. We must refer to the original paper for the tables thus constructed, contenting ourselves with the most prominent results.

Emetics in the first period were given ten times as often as in the second, and twelve times as often as in the last. Formerly, they were given invariably at the onset of fever, as, according to the prevailing theory of Cullen, they took off the spasm of the extreme vessels. Enemata were prescribed during the last forty years somewhat less than one-half as often as during the first twenty. The period when they were most seldom ordered was about the time when Dr. Hamilton's purging practice was introduced. Their use is now becoming more frequent. Local bleeding has made a gradual and steady progress. Blisters and warm plasters have diminished, and the latter have been partly superseded by stimulating liniments, and other modes of counter-irritation. Tartar emetic was given constantly as an emetic in the first period: but, except as an emetic, it is met with most frequently in the third. Kermes mineral was formerly much used in pneumonia: it has ceased to be employed, but the antimonial and James's powder have increased. The total number of the preparations of antimony is nearly equal in each of the three periods. Opium has kept its ground steadily: its camphorated tincture has fallen almost into disuse. Hyoscyamus is more used, owing to the extract being commonly prescribed in conjunction with blue pill. In the first period the red peruvian bark was used generally, and in powder. After that, the infusion and tincture of the pale bark; and now sulphate of quinine has in great measure superseded all. Epsom salt has been but recently commonly employed, its place having been formerly supplied by Rochelle salt, sal polychrest, and the sulphate of soda. Nitre has fallen into comparative disuse. Blue pill was found seventeen times in the first period, thirty-nine in the second, and 156 in the third; owing to Abernethy's recommendation. The use of mercurial ointment in frictions has much diminished. Jalap has decreased, and rhubarb increased. Ipecacuanha is used much more at present than formerly. Fifty years ago blisters were seldom dressed with simple ointment, or allowed to heal soon; basilicon or an ointment of trifolium melilotus was employed. The following medicines have undergone a considerable change:

| | | | |
|---|---|---|---|
| Guaiacum ............ | 32 | 10 | 2 |
| Camphor ............ | 9 | 22 | 52 |
| Colocynth ............ | 19 | 31 | 67 |

*The Dublin Journal, September*, 1836.

---

*Statistics of Suicide in Aberdeen.* By F. Ogston, M.D.

In Aberdeen, out of a population of about 58,000, there have been, in ten years, thirty-eight cases of suicide, and fourteen serious but unsuccessful attempts at suicide. Of the whole number, precisely one-half were of each sex; but, of the successful attempts, twenty-two were males and only sixteen females. The age at which the greater number both of attempted and actual suicides took place, and in both sexes, was from twenty to thirty. As might have been expected, the more violent means, such as cutting instruments and firearms, were had recourse to by males; while poison and drowning were the modes chiefly chosen by females. The three great causes were (1) *Insanity* in some of its forms, (2) *Love disappointments*, and (3) *Family quarrels;* out of the whole fifty-two cases, twenty being from the first, eleven from the second, and eight from the third. Of the eleven cases from love disappointments, eight were females. Out of the twenty-six women, no fewer than eleven were prostitutes. Of thirty of the individuals, not fewer than twenty were intoxicated before attempting suicide; seventeen had the character of habitual drunkards, and an equal number were reputed as temperate. In six of the cases, the attempt was the second; if one, it was the third.

*Medical Gazette, November* 5, 1836.

---

*Statistics of the London Hospital, with remarks on the Law of Sickness.* By T. R. Edmonds, Esq. B.A.

This is the continuation of a former article published by the author in the same journal in February last, and, as well as that, is a most important document. The nature of the article precludes the possibility of giving an intelligible abstract of it; and we cannot give it entire; but we earnestly recommend its perusal to our statistical readers. As a mere *specimen* of its value, we extract a single passage, which points out a most momentous omission in almost all medical reports hitherto published, from which conclusions respecting treatment have been deduced.

"The merit of discovering some peculiarly efficacious treatment of a particular malady is frequently claimed by different medical men, on the ground of the mortality among their patients being unusually low. All mention of the *ages* of their patients is omitted, and no suspicion appears to be entertained of the fact that, under the same medical treatment, a difference of twenty-three years in the ages of the two classes of patients will cause a doubling of the mortality. When the ages of the patients are unknown, the diminished duration of sickness, also, is no ground for presuming on any superiority of treatment. There exists satisfactory (though indirect) evidence that the mean duration of an attack of sickness is equally dependent on the ages of the patients."

[A striking illustration of the truth and importance of this element of *age* in medico-statistical details, will be seen in the abstract given of Dr. Mateer's report of the Belfast Fever Hospital (see p. 260 of our present number); as the results there recorded are in perfect accordance with Mr. Edmonds's principle, although it does not appear that the author of the Report was aware of its influence.]

*Lancet, November* 3, 1836.

